# Efficacy and Safety of the Topical Gene Therapy Beremagene Geperpavec‐svdt (B‐VEC) in an Open‐Label Study of Japanese Subjects With Dystrophic Epidermolysis Bullosa

**DOI:** 10.1111/1346-8138.17863

**Published:** 2025-07-16

**Authors:** Ken Natsuga, Daisuke Tsuruta, Shota Takashima, Chiharu Tateishi, Masaaki Takatoku, Brittani Agostini, Sarrah Mailliard, Nicholas J. Reitze, Rebecca T. Beacham, Alexia M. Cardiges, Michael J. Johnston, Ramakrishna Edukulla, Suma M. Krishnan

**Affiliations:** ^1^ Department of Dermatology Graduate School of Medicine, Hokkaido University Sapporo Japan; ^2^ Department of Dermatology Graduate School of Medicine, Osaka Metropolitan University Osaka Japan; ^3^ Krystal Biotech, Inc. Pittsburgh Pennsylvania USA

**Keywords:** B‐VEC, clinical trial, dystrophic epidermolysis bullosa, Japan, topical therapy

## Abstract

Dystrophic epidermolysis bullosa (DEB) patients have pathogenic variants in *COL7A1*, leading to skin fragility, blistering, and scarring. Beremagene geperpavec‐svdt (B‐VEC) is a replication‐defective herpes simplex virus type 1 (HSV‐1)‐based gene therapy vector administered topically to deliver functional *COL7A1* to DEB wounds. In a United States (US) Phase 3 study, B‐VEC significantly improved wound healing at 3 and 6 months compared to placebo, and in a US open‐label extension (OLE) study, weekly B‐VEC administration was well tolerated for up to 112 weeks. The present study was conducted to confirm the efficacy and safety of B‐VEC in a cohort of Japanese DEB patients receiving weekly B‐VEC treatment (4.0 × 10^9^ plaque forming units (PFU)/mL) for 52 weeks. Wound healing assessments were conducted on a Primary Wound at visits corresponding to Month 3 (the secondary efficacy endpoint), Month 6 (the primary efficacy endpoint), and Months 9 and 12 (exploratory durability endpoints). Patient‐reported outcome (PRO) measures were employed as exploratory analyses of efficacy. Safety was assessed by adverse events (AEs) and clinical laboratory tests. Five subjects enrolled in the study; one discontinued due to challenges with following wound dressing disposal guidelines. The study met its primary and secondary efficacy endpoints with 100% of Primary Wounds demonstrating complete closure at Months 6 and 3, respectively; durable complete closure was observed in 3/4 (75%) of wounds at Months 9 and 12. PROs indicated decreased pain, improvement in skin‐specific quality of life, and moderate to high treatment satisfaction. Four subjects reported ten AEs; all were assessed as mild or moderate in severity and unrelated to treatment by Investigators. None were serious, severe, or led to treatment/study discontinuation. The results of the Japan OLE study are in agreement with the US Phase 3 and OLE studies, demonstrating the efficacy and safety of B‐VEC in Japanese patients with DEB.

**Trial Registration:** Japan Registry of Clinical Trials: jRCT2053230075

## 1 Introduction

Dystrophic epidermolysis bullosa (DEB) is a rare genetic mechanobullous disorder characterized by skin and mucosal membrane fragility, extensive blistering in response to minor trauma, milia formation, and scarring [1]. DEB results from pathogenic variants in the *COL7A1* gene, encoding the structural protein type VII collagen (COL7) [2]. COL7 proteins aggregate into anchoring fibrils, structures of the basement membrane that promote integrity in skin and other mucosal tissues [3, 4]. The lack of functional COL7 in DEB patients causes debilitating blisters and open wounds that can affect large areas of the body, including within mucosa such as the oropharynx, esophagus, and rectum [5]. Not only are these wounds painful, but damage to the oral mucosa and esophagus can lead to chronic malnutrition and stunted development, while extensive scarring can fuse the fingers and toes and cause vision impairment [6, 7, 8]. DEB is inherited in an autosomal recessive (RDEB) or autosomal dominant (DDEB) pattern, with RDEB typically presenting with more severe phenotypes [5]. The global prevalence is estimated between 3 and 15 cases per one million people [9] with estimates in the Japanese population roughly similar at 1–9 in one million [10].

In Japan, the Ministry of Health, Labor, and Welfare have classified DEB as an intractable disease, derived from the Japanese word ‘nanbyo,’ and referring to rare diseases that have resulted mainly from unknown causes or diseases that lack clearly established curative treatments [11]. As such, treatment options in Japan are largely aimed at managing the symptoms of DEB and not at addressing the underlying mechanism of absent functional COL7. The patient advocacy group DebRA Japan recommends avoiding damaging stimuli, protecting wounds with dressings, and managing nutrition, but states that no curative treatments are currently available to the Japanese DEB population [12]. The only approved treatment option is cultured epidermal autografts, also called autologous cultured epidermis or JACE (Japan Tissue Engineering Co. Ltd), which involve a skin biopsy that is taken from the patient, cultured until keratinocytes grow to confluence, and then grafted to the patient's wound [13]. These autografts were initially covered by Japanese public healthcare insurance for severe burn injuries and giant congenital melanocytic nevi, but were also approved for DEB patients in 2019 [14, 15].

The severity of the disorder and the lack of effective treatment options prompted the development of minimally invasive therapies to correct the genetic defect underlying DEB. The first corrective DEB treatment to receive United States (US) Food and Drug Administration (FDA) approval was beremagene gerperpavec‐svdt (B‐VEC; sold commercially as VYJUVEK, Krystal Biotech Inc., Pittsburgh, PA, USA), a topical gene therapy that delivers *COL7A1* directly to DEB wounds. B‐VEC is a modified, non‐replicating herpes simplex virus type 1 (HSV‐1)‐based vector that encodes two copies of wild‐type *COL7A1*. A randomized, open‐label, placebo‐controlled Phase 1/2 trial (GEM‐1; US Clinical Trials Registry NCT03536143) demonstrated molecular correction and wound closure in B‐VEC‐treated wounds [16], supporting the initiation of a double‐blind, intrapatient randomized, placebo‐controlled Phase 3 trial (GEM‐3; NCT04491604). The Phase 3 study included 31 patients (aged 1–44 years) who were treated weekly for 26 weeks (6 months) with either B‐VEC or placebo on matched wound pairs [17]. At 3 months, within the intention‐to‐treat (ITT) population (i.e., all patients whose Primary Wounds underwent randomization, regardless of whether B‐VEC or placebo was applied), 71% of B‐VEC‐treated wounds and only 20% of placebo‐treated wounds showed complete wound healing; this was maintained at 6 months, with 67% complete wound healing for B‐VEC‐treated wounds and 22% for placebo‐treated wounds. The only treatment‐related adverse event (AE), erythema in one subject, was mild and resolved. B‐VEC was well tolerated, and no subjects stopped treatment due to AEs. Among the 31 subjects in the Phase 3 study, four were Asian (non‐Japanese) and two were of Asian and White descent. While the number of Asian participants was too small for statistical analyses, treatment responses among those six subjects generally demonstrated more consistent wound closure of B‐VEC‐treated wounds than placebo‐treated wounds.

A long‐term, open‐label extension (OLE) study (NCT04917874) was initiated in US subjects to provide extended access to B‐VEC to Phase 3 subjects, enroll new subjects naïve to treatment, and collect additional health outcomes [18]. Forty‐seven subjects enrolled, 24 that rolled over from the Phase 3 study and 23 that were treatment‐naïve; subjects were treated weekly for up to 112 weeks. The results of the OLE study provided additional safety data in a broader population and did not change the conclusions regarding tolerability from any of the previous clinical studies. Patient‐reported outcome (PRO) measures were consistent with positive treatment outcomes, including high treatment satisfaction and continued responsiveness to treatment. The present study is an OLE study in Japan conducted to verify the efficacy and safety of B‐VEC in patients of Japanese descent who suffer from DEB.

## 2 Methods

### 2.1 Study Design and Participants

Five patients were enrolled in an OLE study of B‐VEC at two centers in Japan in accordance with the Declaration of Helsinki and Good Clinical Practice guidelines. All five subjects had a clinical diagnosis of DEB. Patients were treated with B‐VEC at a maximum weekly exposure level of 4.0 × 10^9^ plaque‐forming units (PFU)/mL, as all subjects were over the age of three. B‐VEC was applied using a drop matrix technique to create an approximately 1 cm‐by‐1 cm grid. After treatment of a designated Primary Wound, any remaining volume may have been utilized across up to six Secondary Wounds selected during the study. A wound was not treated after it closed unless it re‐opened, in which case it was prioritized for treatment. Study visits occurred weekly for up to 52 weeks, with mandatory visits from Weeks 1–26 and more flexibility from Weeks 27–52 (with the exception of visits on Weeks 36, 38, 40, 48, 50, and 52, corresponding to wound healing assessments). A safety follow‐up (SFU) visit was conducted 90 ± 7 days after the last dose of B‐VEC. All subjects were included in safety analyses; only subjects who completed the study without major protocol deviations were included in efficacy analyses. Patients were not given placebo in this study because of the efficacy of B‐VEC observed in the Phase 3 study.

### 2.2 Efficacy Assessments

Primary Wounds were assessed as open or closed, defined as 100% complete closure of the wound, specified as skin re‐epithelialization without drainage, as assessed live by the Investigator at Week 1 (baseline), Weeks 8, 10, and 12 (corresponding to the 3 month secondary efficacy endpoint), Weeks 22, 24, and 26 (corresponding to the 6 month primary efficacy endpoint), Weeks 36, 38, and 40 (corresponding to the 9 month durability exploratory endpoint), and Weeks 48, 50, and 52 (corresponding to the 12 month durability exploratory endpoint). Primary Wounds were also assessed at the SFU visit. Secondary Wounds were not formally assessed for closure. A Primary Wound was considered closed at 6 months if it was assessed as closed at Week 22, 24, or 26; likewise, a Primary Wound was considered closed at 3 months if it was assessed as closed at Week 8, 10, or 12; at 9 months if it was assessed closed at Week 36, 38, or 40; and at 12 months if it was assessed closed at Week 48, 50, or 52. To demonstrate comparability to the Phase 3 study, Primary Wounds had a secondary definition of closure as two consecutive weeks of closure two weeks apart, equating to closure at Weeks 22 and 24 or Weeks 24 and 26 for closure at 6 months and closure at Weeks 8 and 10 or Weeks 10 and 12 for closure at 3 months.

### 2.3 Safety Assessments

Safety assessments included monitoring of AEs, vital signs (temperature, respiratory rate, and heart rate), and clinical laboratory tests (including anti‐COL7 and anti‐HSV‐1 antibody status). Only treatment‐emergent AEs (TEAEs) were collected. TEAEs were classified by severity (mild, moderate, severe, or life‐threatening) and by relationship to treatment (not related, unlikely related, possibly related, or related), as assessed by the Investigator based on their clinical experience in DEB patient care and the subject's medical history. TEAEs were coded using the Medical Dictionary for Regulatory Activities (MedDRA) version 26.1 system organ classes and preferred terms. Each TEAE was counted once for each subject unless it resolved and recurred. Immunologic evaluation included testing for antibodies against HSV‐1 and COL7, as assessed by a validated plaque reduction neutralization test (PRNT) or enzyme‐linked immunosorbent assay (ELISA), respectively. The ELISA used was commercially available (Euroimmun, Leubeck, Germany) and specifically measured IgG anti‐COL7 antibodies. Patient serum was collected at baseline (Week 1) and Week 26 for pre‐ and post‐exposure analysis.

### 2.4 PROs

Four PROs were collected to assess treatment satisfaction, quality‐of‐life, and pain severity associated with dressing changes. The Treatment Satisfaction Questionnaire for Medication 9 (TSQM‐9) measures patient satisfaction with treatment through nine items that encompass Convenience, Effectiveness, and Global Satisfaction [19, 20]. Domain scores range from 0 to 100, with higher scores representing higher satisfaction in that domain. The TSQM‐9 was completed by four subjects; subjects ≥ 18 years old completed the assessment themselves, while subjects < 18 had a guardian complete it. The TSQM‐9 was administered at the Week 26 and 52 visits.

The second PRO measure, the Skindex‐29, includes 29 items that address the Symptoms, Emotions, and Functioning aspects of skin disease on quality‐of‐life [21, 22]. Subject responses are transformed to a linear scale of 100, with 0 indicating no effect and 100 indicating effect experienced all the time. It was completed by four subjects (all ≥ 12 years old) at the Week 1, 26, 38, and 52 visits.

The third PRO tool, the visual analog scale for pain (VAS Pain), is a measure of pain severity [23]. Subjects indicate on a scale of 0 (no pain) to 10 (worst pain possible) the intensity of pain associated with the Primary Wound following removal of dressings. It was completed by four subjects (all ≥ 6 years old) at the Week 1, 22, 24, and 26 visits.

The fourth and final PRO measure is the EuroQol 5‐Dimension (EQ‐5D), which is comprised of a short descriptive system questionnaire with five levels (EQ‐5D‐5L; assessing Mobility, Self‐Care, Usual Activities, Pain/Discomfort, and Anxiety/Depression) and a visual analog scale (EQ VAS) that measures general health status [24]. It was completed by four subjects (all ≥ 12 years old) at the Week 1, 26, 38, and 52 visits.

### 2.5 Statistical Analysis

Data were summarized using Version 9.4 of Statistical Analysis Software (SAS) with descriptive statistics (number of participants, mean, standard deviation (SD), median, minimum (min), and maximum (max)) for continuous variables and frequency and percentage for categorical variables. PROs are reported as individual subject score and total mean score ± SD.

## 3 Results

### 3.1 Patients

Demographic and baseline characteristics (including Primary Wound size), as well as overall treatment exposure, for the five subjects are summarized in Table 1 and presented individually in the Supporting Information (Table S1). One subject withdrew from the study after Week 8 due to challenges associated with following guidelines regarding wound dressing disposal. The age of subjects ranged from 12.8 to 68.6 years (median 22.3 years); two subjects were 18 years or younger. All subjects had RDEB and were of Japanese descent, and one subject was male. The size of the Primary Wound at baseline ranged from 1 cm^2^ to 20 cm^2^. Subjects spent a median number of 343 days on treatment (from first dose date to the last dose date), with a median number of doses equal to 24.

**TABLE 1:** Subject demographic characteristics and duration of B‐VEC exposure.

	All subjects (*N* = 5)
Age (years, at the Week 1 visit)	
Mean (SD)	36.2 (25.9)
Median (min, max)	22.3 (12.8, 68.6)
Sex, *n* (%)	
Male	1 (20.0)
Female	4 (80.0)
Age by Category, *n* (%)	
≤ 12 years	0
> 12 and ≤ 18 years	2 (40.0)
> 18 years	3 (60.0)
Race, *n* (%)	
Asian	5 (100.0)
Ethnicity, *n* (%)	
Japanese	5 (100.0)
Primary Wound Area (cm^2^)	
Mean (SD)	7.4 (7.6)
Median (min, max)	6 (1, 20)
Primary Wound Area, *n* (%)	
≤ 5 cm^2^	2 (40.0)
> 5 to ≤ 10 cm^2^	2 (20.0)
> 10 to ≤ 15 cm^2^	0
> 15 to ≤ 20 cm^2^	1 (20.0)
Overall Exposure Duration (days)^a^	
Mean (SD)	290 (133.3)
Median (min, max)	343 (52, 358)
Total Number of Doses Received^b^	
Mean (SD)	28.4 (17.9)
Median (min, max)	24 (8, 52)

Abbreviation: SD, standard deviation.

^a^ Exposure duration: From the first dose date to the last dose date.

^b^ A single dose includes the amount of B‐VEC applied to Primary and/or Secondary wounds.

### 3.2 Efficacy

**FIGURE 1:**
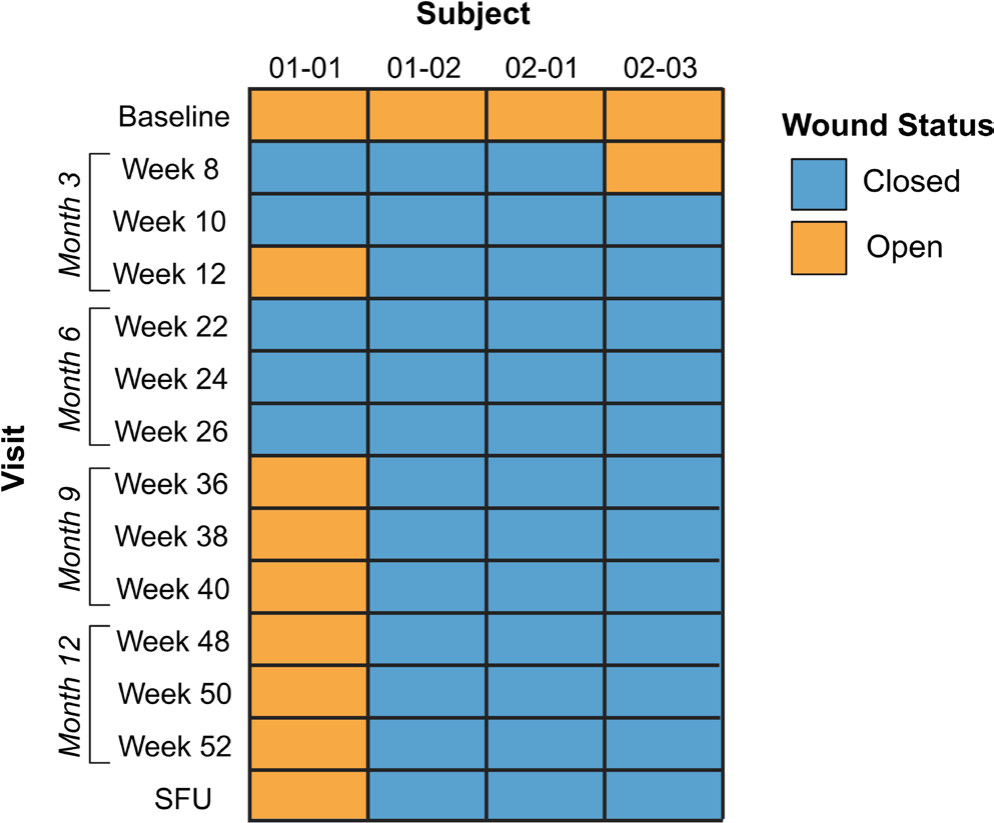
Primary wound closure assessments at baseline, Month 3 (Weeks 8, 10, and 12), Month 6 (Weeks 22, 24, and 26), Month 9 (Weeks 36, 38, and 40), Month 12 (Weeks 48, 50, and 52), and the SFU visit (90 ± 7 days after last dose) for the four subjects that completed the study. Wounds were assessed for 100% complete wound closure, specified as skin re‐epithelialization without drainage, as determined live by the investigator. SFU, safety follow‐up.

**FIGURE 2:**
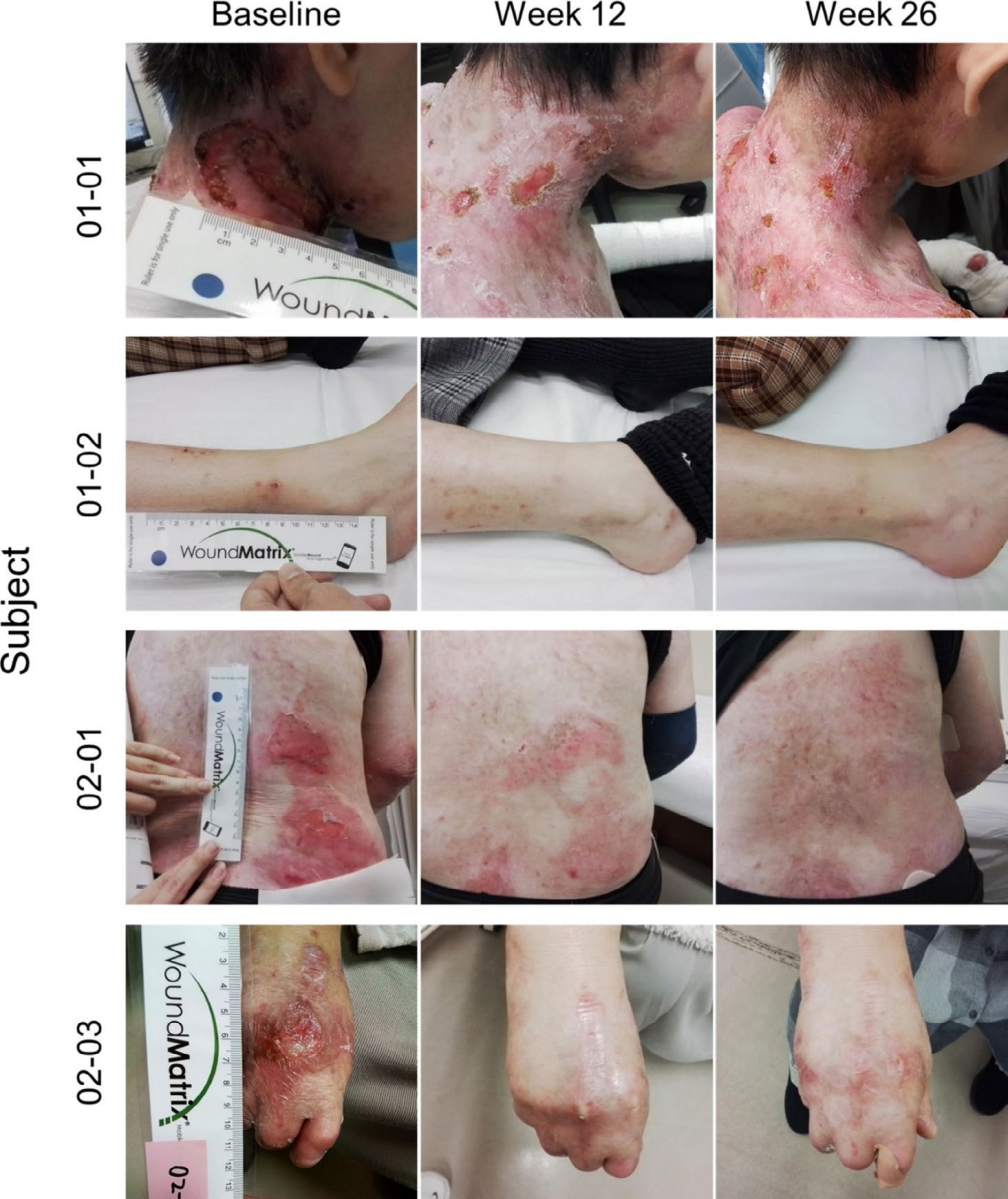
Primary wound images at baseline, Week 12 (Month 3), and Week 26 (Month 6) for the four subjects that completed the study.

Among the four subjects assessed for complete wound closure, 100% (4/4) of Primary Wounds were closed at Week 22, 24, or 26, corresponding to the 6 month primary efficacy endpoint of the study (Figures 1 and 2). These Primary Wounds were also assessed as completely closed at Week 8, 10, or 12, corresponding to the 3 month secondary efficacy endpoint (Figures 1 and 2). As the study was initiated to compare efficacy to the Phase 3 study, a secondary definition of wound closure from the Phase 3 was also utilized, defined as two weeks of consecutive closure spaced two weeks apart between Week 22 & Week 24 or Week 24 & Week 26 for closure at 6 months or between Week 8 & Week 10 or Week 10 & Week 12 for closure at 3 months. Compared to the ITT population from the Phase 3 study, which demonstrated 67% complete wound closure at the Weeks 22 & 24 or Weeks 24 & 26 assessment timepoints [17], the present study demonstrated 100% closure (4/4) based on this definition. Likewise, 71% of the ITT population from Phase 3 met this definition of closure between Weeks 8 & 10 or Weeks 10 & 12 [17], whereas 100% (4/4) of subjects in the present study met this definition, demonstrating comparability between the Japan OLE and Phase 3 studies for wound healing.

Wounds were also assessed at Months 9 and 12 as exploratory measures of durability. B‐VEC promoted the durable complete closure of 3/4 Primary Wounds assessed at Weeks 36, 38, and 40 (Month 9) and Weeks 48, 50, and 52 (Month 12) (Figure 1). Assessment at the SFU visit revealed no change in wound closure status from the Week 52 assessment (i.e., complete closure in 3/4 wounds). Representative images of Primary Wounds at Months 9 and 12 are provided in the Supporting Information (Figure S1).

### 3.3 Safety

**FIGURE 3:**
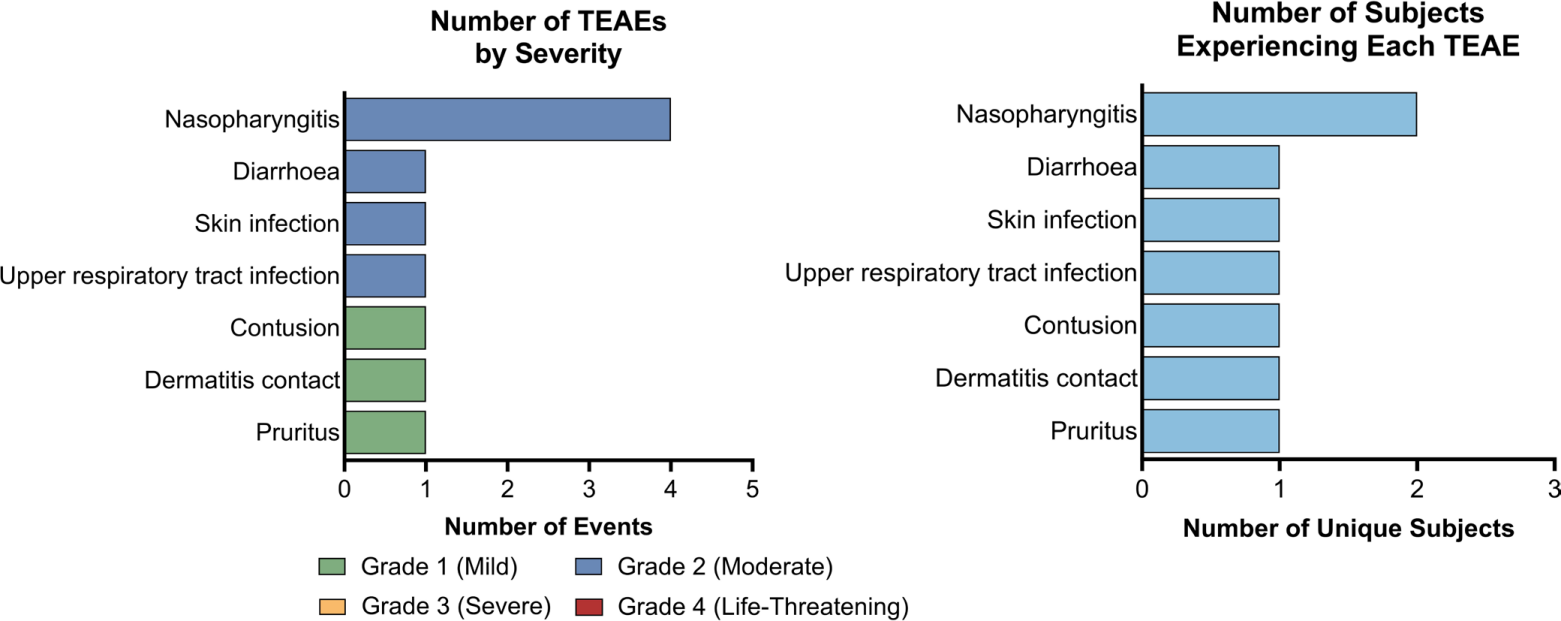
Number of treatment‐emergent adverse events (TEAEs) by severity and the number of unique subjects experiencing each TEAE.

Ten TEAEs were reported by four subjects (4/5; 80%); all were mild or moderate in severity, and none were considered related to B‐VEC treatment by the Investigators (Figure 3). Each TEAE was reported once by a single subject, except nasopharyngitis, which was reported in two subjects and occurred multiple times in the same subject. There were no SAEs and no TEAEs that led to discontinuation. Overall, these AEs resembled those reported during the Phase 3 and US OLE studies [17, 18] and were consistent with common symptoms in the DEB patient population [25].

No clinically significant changes in laboratory values or vital signs were observed during the study. To determine potential immunogenicity, levels of antibodies against HSV‐1 and COL7 pre‐ and post‐treatment were assessed. Due to the difficulty of blood draws in DEB patients owing to skin fragility, only three of the five subjects (60%) were able to provide a serum sample at baseline, and matched serum samples were obtained at Week 26 for two of these subjects. At baseline, all three subjects (3/3) were anti‐HSV‐1 antibody positive, in agreement with seropositivity rates of the general Japanese population [26], and no subjects (0/3) had antibodies against COL7. Of the subjects with matched samples at Week 26, one subject showed no change in HSV‐1 titer and the other showed a slight increase that was not clinically meaningful (as defined by a > 4‐fold sustained increase). Neither subject showed COL7 seroconversion at Week 26.

### 3.4 PROs (Exploratory Efficacy)

**FIGURE 4:**
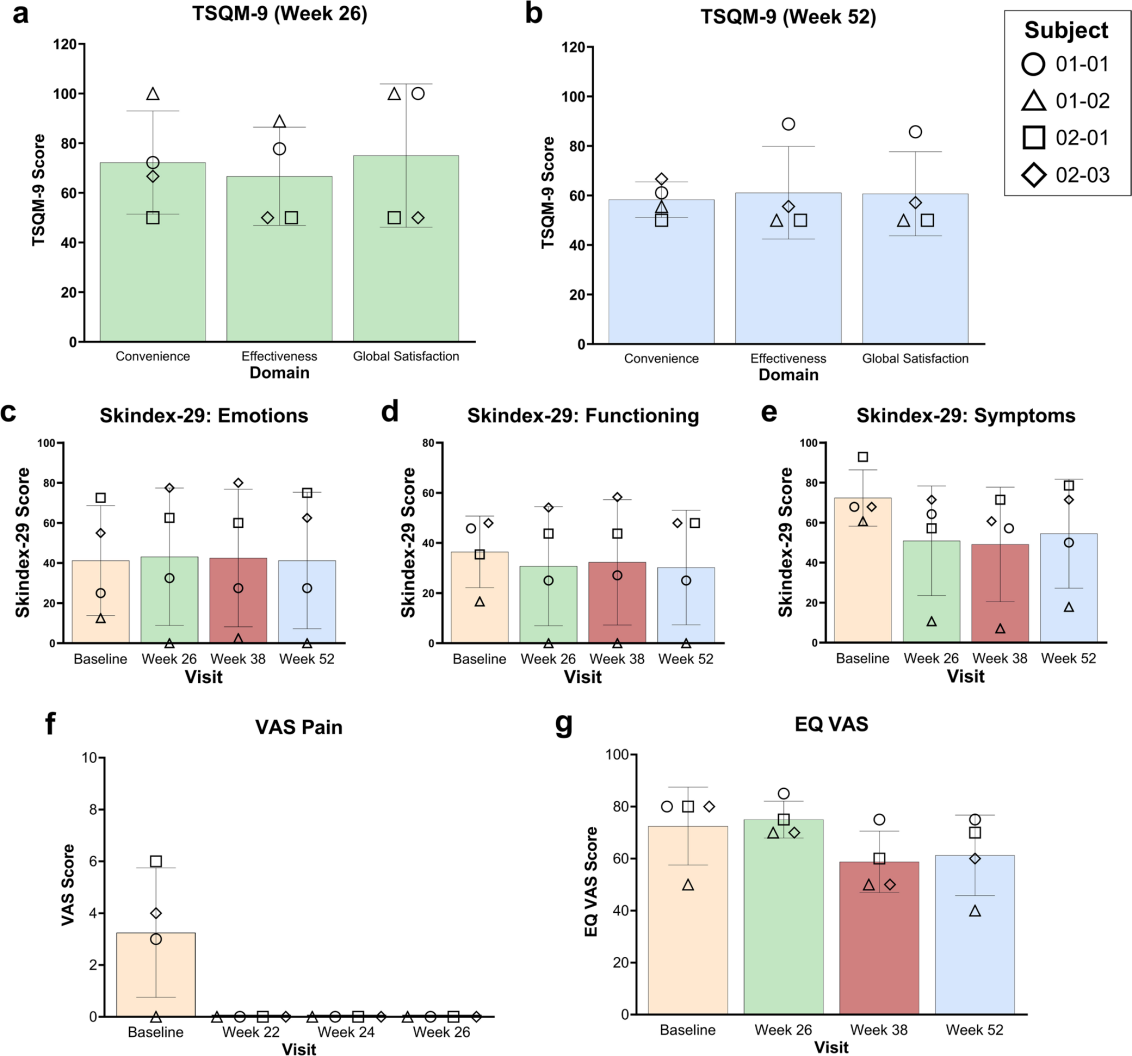
Patient‐reported outcome measures at specific visits during the study. Mean and individual scores are presented at baseline (yellow), Week 26 (green), Week 38 (red), and Week 52 (blue). TSQM‐9 treatment satisfaction is presented for the three domains assessed (Convenience, Effectiveness, and Global Satisfaction) at (a) Week 26 and (b) Week 52. Skindex‐29 (skin‐specific quality‐of‐life) is presented for the (c) Emotions, (d) Functioning, and (e) Symptoms domains. The (f) VAS Pain (severity of pain associated with Primary Wound dressing changes) and (g) EQ VAS (general health status) are presented by visit. EQ VAS, EuroQol visual analog scale; TSQM‐9, Treatment Satisfaction Questionnaire for Medication 9; VAS, visual analog scale.

Four PRO measures were used to assess treatment satisfaction (TSQM‐9), quality‐of‐life (Skindex‐29 and EQ‐5D), and pain severity associated with Primary Wound dressing changes (VAS Pain) as exploratory analyses of efficacy in the four subjects that completed the study. Mean TSQM‐9 scores ± SD were generally high at the Week 26 visit (Convenience = 72.22 ± 20.79, Effectiveness = 66.67 ± 19.77, Global Satisfaction = 75.00 ± 28.87) (Figure 4a) and more moderate at the Week 52 visit (Convenience = 58.34 ± 7.17, Effectiveness = 61.11 ± 18.70, Global Satisfaction = 60.71 ± 17.00) (Figure 4b).

The Skindex‐29 was completed at baseline (Week 1) and Weeks 26, 38, and 52. For the Skindex‐29, a lower score is indicative of less impairment, and thus a decrease from baseline represents improvement. While the small number of subjects prevents statistical significance, there is a downward trend of scores from baseline to Week 52 for the Functioning (36.46 ± 14.28 to 30.21 ± 22.85) (Figure 4d) and Symptoms (72.32 ± 14.10 to 54.46 ± 27.26) (Figure 4e) domains, which is suggestive of improvement in skin‐specific quality‐of‐life, especially for how subjects regarded the symptoms of their DEB after 52 weeks of B‐VEC treatment. The mean Emotions score, however, did not change between baseline and Week 52 (41.25 ± 27.42 to 41.25 ± 34.06) (Figure 4c).

The VAS Pain was completed at baseline (Week 1), and Weeks 22, 24, and 26 as a measure of pain severity associated with removal of the Primary Wound dressing, in which a lower score indicates less pain. Subjects initially reported moderate pain at baseline (mean = 3.25 ± 2.50; range 0 to 6), but all VAS Pain scores for every subject were 0 for Weeks 22, 24, and 26, indicating no pain during Primary Wound dressing changes at these timepoints (Figure 4f). This is suggestive of improved pain outcomes with B‐VEC treatment, correlating with the wound closure observed for all subjects at the Weeks 22, 24, and 26 Primary Wound assessments (Figure 1).

Finally, the EQ‐5D was employed to measure quality‐of‐life. In general, EQ‐5D‐5L responses were inconsistent across the five domains, with the majority of respondents reporting no change in condition (data not shown). The EQ VAS component of the EQ‐5D demonstrated a change in mean score from 72.50 ± 15.00 at baseline to 61.25 ± 15.48 at Week 52 (a difference of −11.25) (Figure 4g). While this decrease in score may be indicative of a deterioration in health status, the large SD and small number of subjects limit the conclusions that can be drawn.

## 4 Discussion

The present study was designed to extend the efficacy and safety of B‐VEC in a cohort of Japanese DEB patients, confirming that no noteworthy differences in treatment response arise from varying ethnic backgrounds. The primary and secondary endpoints of the study, complete closure of the Primary Wound at Months 6 and 3, respectively, were met by 100% (4/4) of subjects; durable complete closure was observed in 75% (3/4) of these wounds assessed at Months 9 and 12. Further supporting these efficacy findings, several PROs suggested decreased wound pain during dressing changes (VAS Pain), improvement in skin‐specific quality of life (Skindex‐29), and moderate‐to‐high treatment satisfaction (TSQM‐9), consistent with wound healing. Evaluation of safety and tolerability of B‐VEC in this population demonstrated only mild and moderate TEAEs, none of which were reported as related to treatment. All TEAEs were consistent with symptoms experienced by the DEB population [25] and were in line with previously reported TEAEs from the Phase 3 and US OLE studies [17, 18]. No clinically significant immunologic reactions, development of anti‐drug antibodies, manifestations of active HSV‐1 infection, or clinically meaningful changes in hematology, serum chemistry, or vitals were observed.

All but one of the subjects demonstrated complete wound closure at Months 9 and 12. The Primary Wound of Subject 01–01 was located on the back of this subject's neck and measured 8 cm^2^ at baseline. The size of this wound did not seem to preclude it from closing, as Subject 02–01, whose Primary Wound was over twice as large at 20 cm^2^ at baseline, demonstrated complete wound closure at every assessed timepoint after baseline. It is possible that the location of Subject 01–01's Primary Wound contributed to the observed re‐opening because this may be a high‐friction area for this subject (e.g., continuous contact with the patient's clothing, such as a shirt collar). While B‐VEC is designed to be topically applied to any skin surface, further studies may provide additional understanding regarding healing rates for different areas of the body.

The interpretation of PRO measures in this study, while generally consistent with wound healing, remains limited by the small number of subjects observed. TSQM‐9 treatment satisfaction scores were generally high (> 65) at Week 26, but more modest (> 55) by Week 52. It is possible that subjects were less enthusiastic regarding treatment in the second half of the study; a potential explanation is that exhaustion from constant travel to the study site caused the lower scores in the Convenience and Global Satisfaction domains. Of note, Subject 01–01, who did not experience closure of the Primary Wound from Weeks 36–52, reported very high scores at the Week 52 visit, including 88.89 for Effectiveness and 85.71 for Global Satisfaction, highlighting the perceived benefits of treatment that subjects may experience even in the absence of 100% wound closure. It was recently reported that reduced TSQM‐9 treatment satisfaction was associated with increased disease severity in a cohort of Japanese prurigo nodularis patients [27], which could offer an explanation for the lower TSQM‐9 scores and EQ VAS scores reported at Week 52. EQ VAS is a measure of general health and is therefore influenced by many aspects of disease burden, including the comorbidities of DEB. Skindex‐29 scores trended towards improvement regarding symptoms and functioning aspects of DEB on skin‐specific quality‐of‐life from baseline to Week 52, while scores for emotions were unchanged. Mean scores for Symptoms and Emotions remained very severe (≥ 52 for Symptoms, ≥ 39 for Emotions), but Functioning scores fell below the cutoff for very severe (≥ 37) and are considered moderate, based on impact on health‐related quality‐of‐life [28]. The largest decrease in score occurred between baseline and Week 26, suggesting that subjects perceived benefits within the first 26 weeks of treatment. However, Week 38 and 52 scores remain similar to Week 26 scores, which may indicate that the largest perceived benefit in treatment occurs soon after starting B‐VEC administration and remains consistent with additional treatment.

Comparison of the PRO measures in the Japan OLE to the Phase 3 [17] and OLE [18] studies reveals similar scores. The TSQM‐9 was not administered during the Phase 3 study but was completed at baseline of the OLE study by subjects that rolled over from Phase 3; it was also completed by treatment‐naïve subjects in the OLE. Japanese subjects reported similar treatment satisfaction to the US subjects from the OLE study. Skindex‐29 scores were also similar to those reported during the Phase 3 and OLE studies. Japanese subjects reported less pain associated with dressing changes of the Primary Wound than was observed in the Phase 3 study (not assessed in US OLE), likely due to the 100% wound closure of all Primary Wounds reported in the present study. The EQ‐5D‐5L was similarly inconsistent when assessed during the Phase 3 and OLE studies. Comparison of the EQ VAS to the Phase 3 and OLE studies is challenging due to the large difference in score at baseline (58.5 ± 25.19 for the modified ITT population in Phase 3; 64.7 ± 18.81 for the safety population in the OLE) compared to the baseline for this study (72.50 ± 15.00). The EQ VAS has been evaluated in a sample of European DEB patients (France, Germany, Italy, Spain, and the United Kingdom), who reported a mean score of 61.9 ± 23.9 for adult patients and 54.8 ± 18.2 for adolescent patients [29]. The mean scores reported for this European cohort are in line with the Phase 3 and US OLE baseline study values but are notably lower than the baseline score reported in Japanese DEB patients in the current study.

When considering B‐VEC treatment, patients and providers should evaluate therapeutic options holistically, including not only the available safety and efficacy data, but also how new treatment paradigms align with current care practices (e.g., the convenience and ease of use within a patient's existing standard of care routine). A survey of DEB patients (*n* = 33) and their caregivers (*n* = 51) reported that 45% and 49%, respectively, spend more than 2 h per day on wound dressing changes [25]. In this trial, following preparation of B‐VEC, it took investigators approximately 5 to 10 min to apply to a wound up to 40 cm^2^. Once weekly application of B‐VEC during dressing changes does not significantly increase the time burden for patients or healthcare workers, allowing patients to not considerably modify their already well‐established wound care routines.

Limitations in the study design include the small cohort of patients enrolled (a necessity due to the nature of DEB as a rare disease) and the lack of control wounds, unlike the Phase 3 study which included a wound matched to the Primary Wound that was administered placebo. Additionally, while both DDEB and RDEB patients were eligible to enroll in the study, only RDEB subjects participated in Japan. However, the efficacy and safety of B‐VEC have been demonstrated in DDEB patients in studies in the US [17, 18], and given the mechanism of action of B‐VEC, no difference in treatment response is anticipated between RDEB and DDEB patients of Japanese descent. Despite these limitations, the present study demonstrated durable wound closure in Japanese DEB patients, with no additional safety signals.

## 5 Conclusions

The results of this study are consistent with the results of the US Phase 3 and OLE studies of B‐VEC, indicating no apparent difference in efficacy or safety for Japanese subjects undergoing treatment spanning 52 weeks. The positive findings of B‐VEC treatment in the US DEB population can be extended to the Japanese DEB population.

## Ethics Statement

This study was performed in accordance with the Declaration of Helsinki, the International Council for Harmonisation of Technical Requirements for Pharmaceuticals for Human Use (ICH) Good Clinical Practice guidelines, and other applicable laws and regulations. Approval was obtained from the Osaka Metropolitan University Hospital IRB and the Hokkaido University Hospital IRB.

## Consent

All participants gave written informed consent/assent to participate in the research. At least one parent or guardian for each adolescent patient provided written informed consent.

## Conflicts of Interest

Ken Natsuga has received grants from Japan Tissue Engineering Co. Ltd. Daisuke Tsuruta is an Editorial Board member of *Journal of Dermatology* and a co‐author. To minimize bias, he was excluded from all editorial decision‐making related to the acceptance of this article for publication. Ken Natsuga and Daisuke Tsuruta have received advisory and lecture fees from Krystal Biotech Inc. Masaaki Takatoku, Brittani Agostini, Sarrah Mailliard, Nicholas J. Reitze, Rebecca T. Beacham, Alexia M. Cardiges, Michael J. Johnston, Ramakrishna Edukulla, and Suma M. Krishnan are employees and shareholders of Krystal Biotech Inc. Shota Takashima and Chiharu Tateishi have no conflicts to declare.

## Supporting information


Figure S1.

Data S1.

## Data Availability

The data that support the findings of this study are available from the corresponding author upon reasonable request.
