# Supplementary figures and images for: Efficacy and Safety of the Topical Gene Therapy Beremagene Geperpavec‐svdt (B‐VEC) in an Open‐Label Study of Japanese Subjects With Dystrophic Epidermolysis Bullosa
Source: J Dermatol. 2025 Jul 16;52(10):1494–502. doi: 10.1111/1346-8138.17863 (PMC12530466; doi:10.1111/1346-8138.17863)

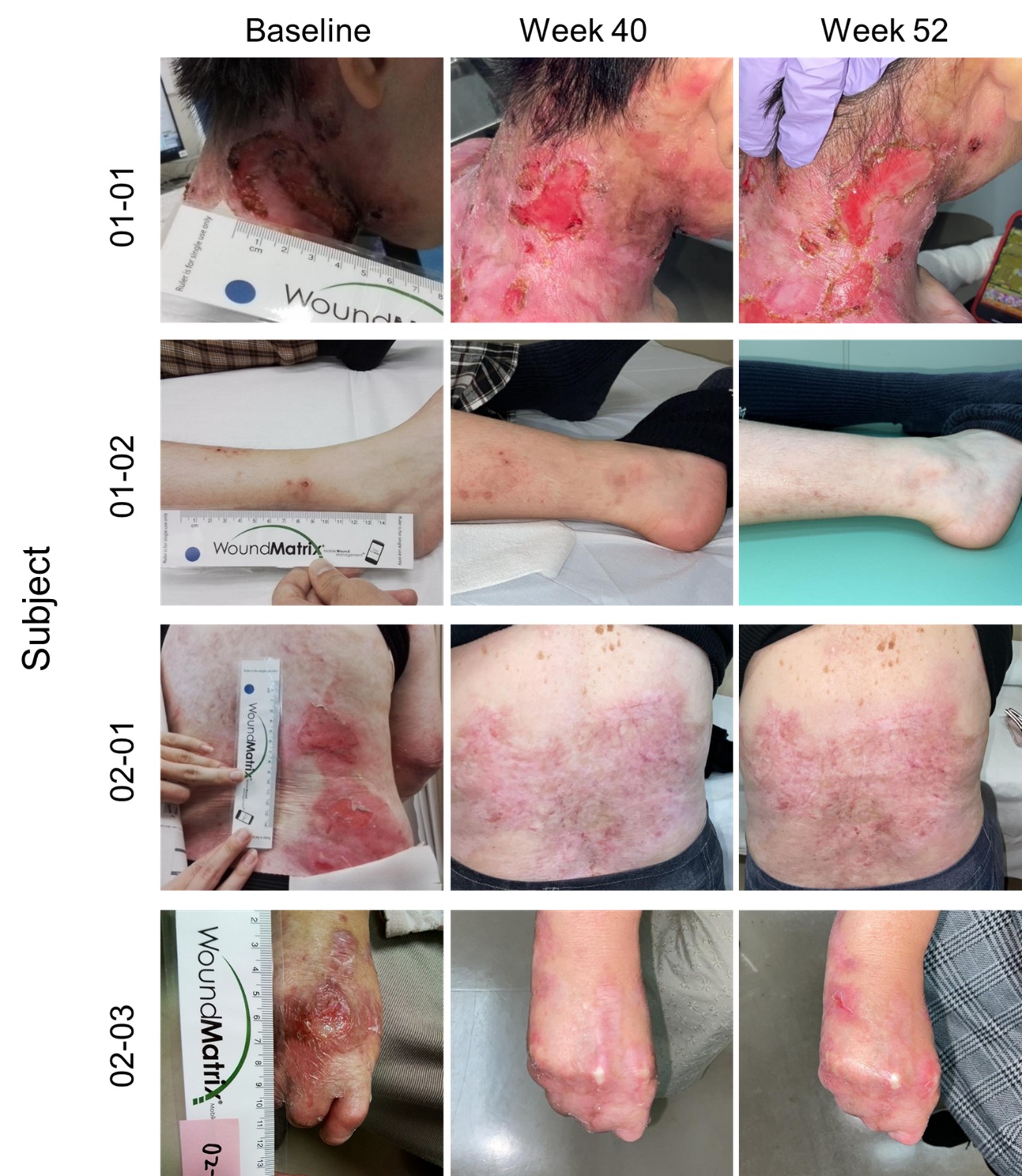

Supplement: Supplementary file 1 — Figure S1. [file JDE-52-1494-s001.jpg]
