# Supplementary material for: Efficacy and Safety of the Topical Gene Therapy Beremagene Geperpavec‐svdt (B‐VEC) in an Open‐Label Study of Japanese Subjects With Dystrophic Epidermolysis Bullosa
Source: J Dermatol. 2025 Jul 16;52(10):1494–502. doi: 10.1111/1346-8138.17863 (PMC12530466; doi:10.1111/1346-8138.17863)
Supplement: Supplementary file 2 — Data S1. [file JDE-52-1494-s002.docx]

Supporting Information

Table S1. Individual Subject Demographic Characteristics and Duration of B-VEC Exposure

| **Subject** | **Age (Years)^*^** | **Sex** | **Genotype** | ***COL7A1* Variants**^†^ | **Primary Wound Baseline Size (cm^2^)** | **Overall Exposure Duration (days)**^‡^ | **Total Number of Doses Received**^§^ |
| --- | --- | --- | --- | --- | --- | --- | --- |
| 01-01 | 17.7 | M | RDEB | c.5819delC;  p.R2063W | 8 | 358 | 41 |
| 01-02 | 12.8 | F | RDEB | c.4207G>A (p.G1403S); c.6573+1G>C | 1 | 343 | 24 |
| 02-01 | 68.5 | F | RDEB | c.5443G>A (p.G1815R); c.5819delC | 20 | 354 | 52 |
| 02-02 | 22.3 | F | RDEB | c.130G>A (p.D44N);  c.409C>T (p.R137X) | 6 | 52 | 8 |
| 02-03 | 59.7 | F | RDEB | c.5604 +1G>A;  c.8569G>T (p.E2857X) | 2 | 343 | 17 |

^*^Age is calculated as (date of informed consent – date of birth) / 365.25 ^†^Sequencing notations are reported as recorded in the subject’s medical history.
^‡^Exposure duration: From the first dose date to the last dose date.
^§^A single dose includes the amount of B-VEC applied to Primary and/or Secondary wounds.


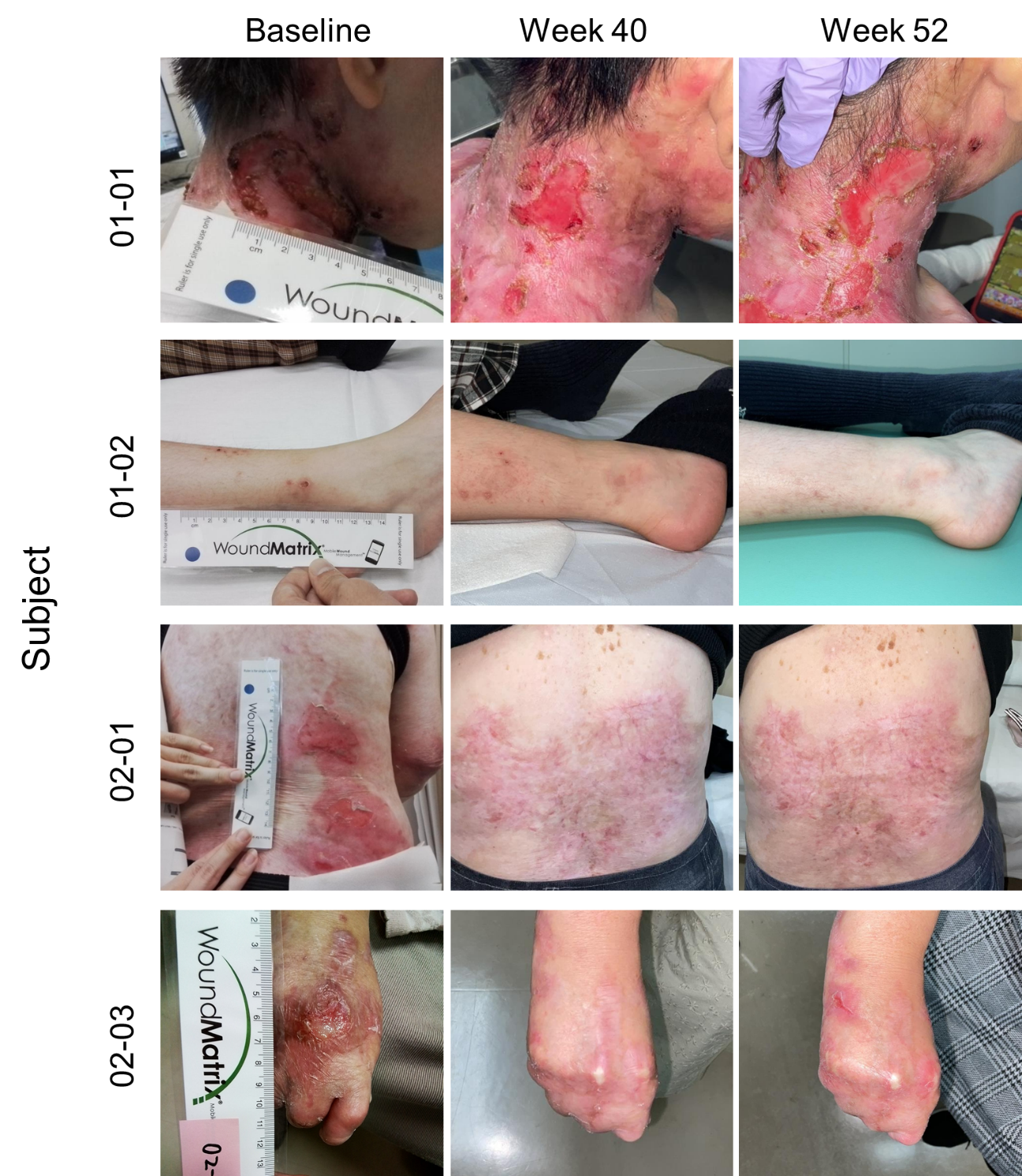


**Figure S1.** Primary Wound images at baseline, Week 40 (Month 9), and Week 52 (Month 12) for the four subjects that completed the study.
